# Episodic memory and self-reference via semantic autobiographical memory: insights from an fMRI study in younger and older adults

**DOI:** 10.3389/fnbeh.2014.00449

**Published:** 2015-01-13

**Authors:** Sandrine Kalenzaga, Marco Sperduti, Adèle Anssens, Penelope Martinelli, Anne-Dominique Devauchelle, Thierry Gallarda, Marion Delhommeau, Stéphanie Lion, Isabelle Amado, Marie-Odile Krebs, Catherine Oppenheim, Pascale Piolino

**Affiliations:** ^1^Memory and Cognition Laboratory, Institut de Psychologie, Université Paris Descartes, Sorbonne Paris CitéBoulogne-Billancourt, France; ^2^Center of Psychiatry and Neurosciences, INSERM UMR S894, Université Paris DescartesParis, France; ^3^Research Center in Cognition and Learning, UMR-CNRS 7295, Université de PoitiersPoitiers, France; ^4^Department of Radiology, Centre Hospitalier Sainte-Anne, Université Paris Descartes, Sorbonne Paris CitéParis, France; ^5^Laboratory of Physiopathology of Psychiatric Diseases, Centre Hospitalier Sainte AnneParis, France; ^6^Institut Universitaire de FranceFrance

**Keywords:** self-reference effect, autobiographical memory, semantic memory, source memory, autonoetic consciousness, medial prefrontal cortex, aging

## Abstract

Self-referential processing relies mainly on the medial prefrontal cortex (MPFC) and enhances memory encoding (i.e., Self-Reference Effect, SRE) as it improves the accuracy and richness of remembering in both young and older adults. However, studies on age-related changes in the neural correlates of the SRE on the subjective (i.e., autonoetic consciousness) and the objective (i.e., source memory) qualitative features of episodic memory are lacking. In the present fMRI study, we compared the effects of a self-related (semantic autobiographical memory task) and a non self-related (general semantic memory task) encoding condition on subsequent episodic memory retrieval. We investigated encoding-related activity during each condition in two groups of 19 younger and 16 older adults. Behaviorally, the SRE improved subjective memory performance in both groups but objective memory only in young adults. At the neural level, a direct comparison between self-related and non self-related conditions revealed that SRE mainly activated the cortical midline system, especially the MPFC, in both groups. Additionally, in older adults and regardless of the condition, greater activity was found in a fronto-parietal network. Overall, correlations were noted between source memory performance and activity in the MPFC (irrespective of age) and visual areas (mediated by age). Thus, the present findings expand evidence of the role of the MPFC in self-referential processing in the context of source memory benefit in both young and older adults using incidental encoding via semantic autobiographical memory. However, our finding suggests that its role is less effective in aging.

## Introduction

Healthy aging is associated with significant changes in episodic memory, defined as the memory of specific episodes. In particular, age-related deficits are pronounced for the qualitative aspects of memories, namely the ability to relive a previous event in a subjective way (i.e., autonoetic consciousness; Parkin and Walter, 1992; Perfect and Dasgupta, 1997; Clarys et al., 2002; Piolino et al., 2006), and for contextual details, i.e., the ability to objectively retrieve the encoding context (e.g., source memory; Spencer and Raz, 1995 for reviews; Mitchell and Johnson, 2009). Older adults have some difficulties in determining the context or experimental encoding conditions of a previously encountered item (Johnson et al., 1993 for a review). These age-related impairments in source memory are particularly pronounced when encoding contexts are very similar (Hashtroudi et al., 1989; Henkel et al., 1998).

Source memory may rely on strategic processes (Fletcher and Henson, 2001; Dobbins and Han, 2006) that are deficient in older adults (Johnson et al., 1993) Thus, it has been proposed that one way to reduce age-related deficits in source monitoring is to give encoding instructions that favor the link between an item and its context, for example by asking participants to decide to what extent an item suits its encoding context (Hashtroudi et al., 1994; Glisky et al., 2001; Naveh-Benjamin et al., 2007; Glisky and Kong, 2008). The encoding strategy that has been considered as the most effective for a few decades is self-referential processing that consists of implicitly or explicitly linking the information to be remembered with personal knowledge. There are mainly two self-reference encoding tasks: those requiring participants to decide if a word to be remembered describes their personality, and those requiring participants to retrieve an autobiographical memory related to the word to be remembered. The latter task has received much less attention to date (Klein et al., 1989). The enhanced memory for self-referenced information is known as the “Self-Reference Effect” (SRE; Rogers et al., 1977; for a review, see Symons and Johnson, 1997). Self-referential encoding (incidental or intentional) gives rise to a mnemonic advantage since this kind of processing promotes organization and elaboration of the material to be remembered (Rogers et al., 1977; Klein and Kihlstrom, 1986). The SRE using nouns is typically obtained with self-reference tasks promoting autobiographical memory retrieval, while the SRE using adjectives is found with tasks requiring participants to describe their personality (for reviews, see Symons and Johnson, 1997; Klein, 2012).

Previous research in young adults has demonstrated that the SRE improves both factual and contextual remembering, notably source memory (Serbun et al., 2011; Leshikar and Duarte, 2012), even with incidental encoding (Lalanne et al., 2013). Moreover, self-referenced items are recollected more vividly than items processed semantically (Dewhurst and Conway, 1994; Conway et al., 2001; Fujita and Horiuchi, 2004) or with reference to other people (Conway and Dewhurst, 1995). This phenomenon has been mainly demonstrated using the Remember/Know/Guess procedure in which participants are asked to state the nature of their recollective experience during free recall or recognition tasks (Tulving, 1985; Gardiner, 1988). This procedure makes it possible to differentiate noetic consciousness (i.e., knowing that an item occurs with no trace of specific detail) from autonoetic consciousness (i.e., remembering that an item occurs with some trace of specific details). Thus, self-reference encoding improves the objective as well as the subjective characteristics of episodic memory.

Due to the beneficial effect of self-referential processing on both factual and contextual episodic memory features, it has been applied in healthy aging as a strategy to reduce age-related episodic memory impairments. Findings have indicated that the SRE is still effective in aging, increasing the amount of items retrieved similarly to young adults. Furthermore, it is robust across encoding (e.g., incidental or intentional learning), and testing tasks (e.g., recall or recognition), as well as across different comparison conditions (e.g., semantic processing or processing with respect to other people) (Mueller et al., 1986; Gutchess et al., 2007, 2010; Glisky and Marquine, 2009; Kalenzaga et al., 2012; Kalenzaga and Clarys, 2013; Lalanne et al., 2013). Moreover, elderly people may benefit from self-reference encoding in the enhancement of both memory for specific details and source memory (Dulas et al., 2011; Hamami et al., 2011; Lalanne et al., 2013). However, even if the SRE exhibited by older adults in these studies is approximately the same size as that found in younger adults, their overall level of performance still remains lower.

Until now, few studies have addressed the question of the neural basis of the SRE on memory specificity in aging. Compared to non-self processing, self-referential processing has been associated with greater activity in cortical midline structures (CMS), a set of brain regions located on the median wall of the frontal and the parietal cortices, comprising the medial prefrontal cortex (MPFC), the anterior cingulate cortex (ACC), and the posterior cingulate cortex (PCC), (Craik et al., 1999; Kelley et al., 2002; Kjaer et al., 2002; Fossati et al., 2003; Lou et al., 2004; Northoff and Bermpohl, 2004; D’Argembeau et al., 2005; Heatherton et al., 2006; Northoff et al., 2006; Gutchess et al., 2007; Raposo et al., 2010; Morel et al., 2014). In particular, the MPFC seems to play a pivotal role in self-referential processing (Northoff and Bermpohl, 2004; Denny et al., 2012; Martinelli et al., 2013c for reviews), and the differential engagement of the MPFC during self-referential encoding characterizes later remembered or forgotten items (Macrae et al., 2004; Zhu et al., 2012; Morel et al., 2014). Another study investigating the neural correlates of subsequent recognition (pictures of common objects) and source memory (background scenes) of information learned under either self or non-self-reference encoding instructions reported that the MPFC, and particularly BA10, contributes to the benefit of the SRE on source memory (Leshikar and Duarte, 2012). Moreover, Philippi et al. (2012) reported that a lesion of this structure abolished the SRE, which corroborates the critical role of this region in this effect.

Data from both neuropsychological and neuroimaging studies suggest that normal aging is accompanied by a decline in the functioning of the prefrontal cortex (for a review, see Park and Gutchess, 2005). However, while aging is associated with structural (Raz, 2000) and functional (Cabeza et al., 1997; Cabeza, 2002; Logan et al., 2002; Gutchess et al., 2005; Reuter-Lorenz and Lustig, 2005) changes in a number of lateral frontal areas, CMS, including the MPFC, seem to be resilient in healthy older adults. For example, both young and older adults similarly activated the MPFC when thinking about the self, compared to thinking about another person (Gutchess et al., 2007). This may explain why self-representation is unaffected by aging (Terracciano et al., 2010; Martinelli et al., 2013a), and why self-referential processing is intact in healthy older adults (Gutchess et al., 2007, 2010; Glisky and Marquine, 2009; Kalenzaga et al., 2012; Kalenzaga and Clarys, 2013; Lalanne et al., 2013). Nevertheless, there might be fundamental age differences in the regions linked to successful encoding of self-referenced information with a reduction of specificity in the MPFC. Indeed, Gutchess et al. (2010) established that older adults tend to show subsequent self-referenced memory effects in prefrontal and parietal regions which are related to subsequent forgetting effects in young adults.

The vast majority of self-referential tasks require participants to decide to what extent a set of personality traits describes their personality, tapping one of the most abstract levels of self-representation—conceptual self-knowledge (Conway, 2005; Klein, 2010). Thus, neuroimaging studies on self-referential processing have mostly studied the neural correlates of the SRE involving such conceptual knowledge. To date, the study of self-referential processing involving autobiographical memories is missing. Moreover, given that healthy aging affects only the episodic component of autobiographical memory, sparing the semantic component (Levine et al., 2002; Piolino et al., 2002, 2006, 2010; Martinelli et al., 2013a,b), the SRE via autobiographical processing should be more effective if it is based on semantic autobiographical memories (SAMs, i.e., generic personal memories, decontextualized extended or repeated events) rather than episodic ones (i.e., unique events situated in time and space). In a recent meta-analysis, Martinelli et al. (2013c) reported that SAM is associated with the activation of a large network including the MPFC, confirming the key role of this structure in the retrieval of self-referential information independently of the level of abstraction (see also Cabeza and St Jacques, 2007).

Although self-referential processing is an effective way of improving episodic memory in healthy aging, studies are lacking in this field. In particular, to the best of our knowledge, until now no neuroimaging study has explored the relationship between the SRE via SAM and qualitative aspects of episodic memory. The present study used an “ecological” experimental situation to assess the effect of an *incidental* self-referencing encoding elicited by *semantic autobiographical memory* on subsequent *delayed free recall*. Thus, the aim of the present study was to explore the neural correlates of the SRE affecting the qualitative (i.e., autonoetic consciousness and source memory) aspects of episodic memory traces in groups of young and older adults. To this end, participants’ brain activity was measured by means of fMRI while performing two incidental encoding tasks (i.e., self and non-self related tasks) of verbal cues. We designed an original self-referencing task to tap cue-based SAM retrieval (i.e., generic personal memories).

According to the Self-Memory System’s hierarchy (Conway, 2005, 2009), SAM is the second most abstract system after self-concepts. Thus, we can assume that the well-known generic memories contained in this highly semanticized system should improve the elaboration and organization of the material (Klein and Loftus, 1988). Regarding non-self related processing, we designed a general semantic task consisting of asking participants to complete a sentence with a common word and to imagine the described scene at the North Pole. Outside the scanner, we then asked the participants to recollect each verbal cue seen in the scanner (surprise free recall memory test).

At a behavioral level, we predicted that self-referencing should improve both autonoetic consciousness and source memory in young and older adults (Dulas et al., 2011; Hamami et al., 2011), but that age-related decline would be observed, especially for source memory. At a neural level, during the incidental encoding of self-referenced cues via SAM retrieval (vs. general semantic memory), we expected to replicate previous findings on SAM, reporting activities in a widespread network encompassing CMS, especially the MPFC (Martinelli et al., 2013c). Regarding the age effect, based on the literature focused on SRE, we expected a relative preservation of the regions engaged during self-referential processing with no difference between the two groups in the CMS (Gutchess et al., 2007), but possibly greater activation for older adults compared to young adults in a number of extra CMS areas such as the frontal and parietal cortex which would reflect a reduction of specificity in CMS (Gutchess et al., 2010). Finally, we expected that the activity during the SRE condition in several CMS regions would correlate with subsequent episodic memory recall for both age groups. In particular, based on previous results, we assumed that activity in the MPFC should contribute to the source memory benefit for self-referenced information in older adults, as previously found in young adults (Leshikar and Duarte, 2012). Nevertheless, if there is a loss of specificity in areas related to successful encoding of self-referenced information in aging (i.e., reduction of specificity in the MPFC), we hypothesized that additional age-related correlations would be found between subsequent self-referenced memory effects and extra CMS regions (Gutchess et al., 2010).

## Methods

### Participants

Nineteen healthy young (YA) (25–44 years old, mean = 29.2 ± 5.69, 10 women) and 16 healthy older (OA) adults (65–80 years old, mean = 68.31 ± 4.51, 9 women), all right-handed (according to the Edinburgh Handedness Inventory; Oldfield, 1971) and native French speakers, participated in the study. All participants gave their informed written consent as required by the local ethics committee (CPP Ile de France 3 *n*°2687). All were unmedicated, living at home and in good general health (clinically screened by a medical exam including hypertension and cerebrovascular risk factors with the Fazekas scale). Exclusion criteria included presence of a history of alcohol or substance abuse, head trauma, major disease affecting brain function, neuropsychiatric disorders (tested with the Mini-International Neuropsychiatric Interview, Sheehan et al., 1998), depression (tested with the Depression Scale, Yesavage et al., 1983; 30 items, cut-off score >10; YA: 2.68 ± 2.6 and OA: 3.62 ± 2.65), abnormal general cognitive functioning as assessed by the Mattis scale (Mattis, 1976, cut-off score lower than 136; YA: 142.44 ± 1.19 and OA: 140.06 ± 3.04), and abnormal visual mental imagery ability (short form of Minnesota Paper Form Board: Vandenberg and Kuse, 1978 (lower than 2 points over 5 points); YA: 4.47 ± 0.77 and OA: 4.12 ± 0.8). Moreover, they all performed within their normal age range for memory as assessed by the Grober and Buschke (1987) test (sum of three total recalls, delayed total recall; YA: 47.94 ± 1.77, 16 ± 0.00 and OA: 45.50 ± 3.01, 15.50 ± 0.89). Finally, both age groups were matched according to their verbal abilities and crystallized intelligence as assessed by the Mill Hill test (Deltour, 1993; a multiple-choice synonym vocabulary test), (percentile score; YA: 55.28 ± 17.27 and OA: 58.96 ± 25.69, *t*_(33)_ = −0.34, *p* = 0.73). For the purpose of the study, both age groups were compared on a self-concept scale (French version, see Lalanne et al., 2013; Martinelli et al., 2013a), adapted from the TSCS (Fitts and Warren, 1996). Both age groups were matched for valence (mean score; YA: 300.89 ± 27.84 and AO: 290.80 ± 18.36, *t*_(33)_ = 1.21, *p* = 0.25) and definite sense of the self (mean score; YA: 31.94 ± 9.52 and OA: 33.13 ± 18.00, *t*_(33)_ = −0.25, *p* = 0.80).

### Procedure

#### General organization

This experiment included an incidental encoding task, divided into two conditions, followed by a delayed free recall. Thus, it comprised three major phases. During the first phase (pre-scanning interview), about 1 month before the scanning session, participants were tested for exclusion and inclusion criteria, they underwent a medical examination, a neuropsychological assessment and completed the Taste and Interest Questionnaire (TIQ) that was employed to create personal verbal cues used in the scanning session. In the second phase (scanning session), participants were trained for the experimental task outside the scanner, then executed the task in the scanner. Finally, in the third phase (memory recall) about 20 min after the end of the scanning session, participants were asked to recall outside the scanner as many verbal cues seen during the scanning session as they could.

#### Pre-scanning interview

During the pre-scanning interview, exclusion and inclusion criteria were verified by means of a clinical exam and psychometric tests, followed by neuropsychological tests and the TIQ. The aim of this questionnaire (Sperduti et al., 2013; Martinelli et al., 2013b) was to collect information in order to create personalized cues for each participant without directly asking them to describe past memories to avoid re-encoding of memories (Viard et al., 2007; Addis et al., 2011). Participants were informed that the purpose of the questionnaire was to obtain a description of their personality thanks to information about their principal life interests excluding the last 5 years. Participants had no prior knowledge of the aim of the fMRI task, preventing the possibility of searching for memories linked to their tastes and interests between the two sessions. The questionnaire consisted of a list of 220 interests including leisure, food, drink, sport, transport, residence, holidays, jobs, and studies. For each item (e.g., Chinese food), participants had to answer whether it was personally pertinent or not, rated by 1 and 0 respectively. When an item was pertinent, they had to rate how important (from 0 to 10) and frequent (Frequent/Rare) the activity or interest had been in their life. An activity or interest was used as a cue for SAM retrieval if it was pertinent, important (>5), and frequent (e.g., chess club). Twenty-four personalized cues were created for each subject with the following structure: “a habit of your past linked to…”.

#### Scanning session

Participants were first invited to take part in a training session before the fMRI scanning. They received detailed explanations about the nature of the tasks and participated in a brief simulation of the experiment on a laptop. Two conditions were explained to the subjects: a self-referential condition through SAM retrieval (*self* condition) and a control non-self condition through general semantic scene imagery (*imagery* condition). No mention was made about subsequent recall.

For the self-referential condition, participants were instructed to mentally recall SAMs elicited by the cues under the scan. SAMs were defined as generic memories of a repeated event that occurred several times in the past, a regular activity that used to occur at a routine time and place or a memory of an extended event that could describe a summary of events over several days, weeks, or months without a precise moment in time (e.g., “a habit of your past about tennis”). They were instructed to search and then recall each SAM (i.e., mental images of generic personal events).

The control non-self condition consisted of a sentence completion task (e.g., “the teacher teaches…”) based on the Beauregard verbal automatism test (Beauregard, 1971), which consists in completing familiar phrases, idiomatic expressions, and proverbs. The subjects were instructed to first complete the sentence with the first word that came to mind (e.g., “lessons”) and then to imagine the scene described in the sentence in a peculiar non-self context, the North Pole. This scenario was used due to the non-personal character of the North Pole since we wanted to avoid any reference to autobiographical memory. Thus, participants were explicitly instructed to imagine the scenes with no reference to their personal life. The design of such a control condition aimed at controlling processes involved in the SAM retrieval task but not related to the self: encoding of new information, access to general semantic knowledge, scene construction and mental imagery. Moreover, for both conditions, participants were asked to press a button as soon as they gained access to the generic personal memory (SAM retrieval) or had completed the sentence and begun to visualize the imagined scene in the context of the North Pole. After instructions, participants were trained on three trials for each condition with the experimenter giving feedback concerning the pertinence of each response. The cues used for the trials were different from those used during the scanning session.

During scanning, cues were visually presented in white font on a black background projected on a screen viewed by means of a mirror incorporated into the head coil. E-Prime software (Psychology Software Tools, Inc., Pittsburgh, PA, USA) in combination with the Integrated Functional Imaging System (IFIS) was used for the presentation and timing of stimuli and collection of responses. Responses were made on an MR-compatible two-button box.

Participants completed four functional scans, each lasting 5 min and 20 s, in a single session. Each functional scan was composed of 12 items (6 self and 6 imagery) presented in a randomized order within mini-blocks of three items of the same condition. Each trial lasted 27 s with the following time-course: the cue was presented for 5 s, followed by a white cross at the center of the screen for 19 s, then the cross turned red for 3 s informing the participants of the end of the present trial and the upcoming of the next one.

#### Recall session

After the scanning session, there was a 20-min retention interval during which participants had time to dress, to rest and go to the debriefing room. They were then invited to freely recall all the verbal cues seen in the scanner during the fMRI session and to indicate, for each cue, the nature of recollection and specific contextual information. Participants were instructed about the differences between remembering, knowing and guessing (Tulving, 1985; Gardiner, 1988; Mäntylä, 1993). A Remember response meant that cues’ recall was accompanied by the ability to mentally travel back in time and re-experience something about its presentation in the scanner, i.e., some aspects of the original experience such as seeing the cue on the screen in the scanner. Participants were required to give either a Know response if retrieval was achieved without any such recollection, or a Guess response if retrieval was doubtful. This latter alternative was provided in order to ensure that the Remember or Know responses did not contain any degree of uncertainty. The standard instructions were provided for all three categories of responses and were very carefully explained to the participants until each kind of response was fully understood. Participants were asked to repeat the instructions to make sure they had understood the nature of the task. Only the Remember responses that reflected the correct cues were taken into account.

In order to assess source memory, participants were instructed to indicate in detail the encoding context of each verbal cue recalled, i.e., self or non-self condition (e.g., “I saw a sentence about *tennis*, it was about myself, I pressed the button and remembered my habits about tennis” or “I completed the sentence the teacher teaches with the word *lessons*, I pressed the button and imagined something about it in the North Pole”). Only the responses that accurately reflected the encoding condition were taken into account (e.g., the condition was the right one, the participant had effectively pressed the button indicating SAM retrieval or sentence completion for the cue). We also asked in which functional runs the cue had been presented (1–4). However, this information was not used in subsequent analyses because there was a floor effect in the older group.

Finally, there was a debriefing during which participants were asked to recall again each SAM retrieved during the scanning session in order to check that those memories met the criterion of generic memories (i.e., repeated events that occurred several times in the past without a precise moment in time: “I remembered my tennis lessons when I was in high school, I saw myself doing the trip with my best friend to go to Roland Garros and have fun together”). They were also asked to recall each scene again in order to check for non-self reference (“I imagined a class in an igloo with the teacher and students all bundled up in snowsuits. They sat writing their lessons with mittens”). The subsequent analyses were performed only on cues that met these criteria and for which the participants had pressed the button in the scan indicating the treatment of the cue (i.e., presence of generic memory retrieval or sentence completion and scene imagery).

For each experimental condition, we assessed the participants’ memory performance by computing two scores: the percentage of correctly recollected items (CRI) and the percentage of correctly recalled sources (CRS) as a function of the total number of correct trials by condition (i.e., the number of trials (SAM or scene imagery) for which the participant had pressed the button under the scan, and that met the criterion at debriefing, max. 24).

The percentage of self-referenced CRI and CRS informed us about the participant’s capacity to benefit from the SRE on autonoetic consciousness and source memory, respectively.

### fMRI method

#### fMRI data acquisition

All data were acquired with a 3 T scanner (Discovery MR 750, General Electric Healthcare, Little Chalfont, United Kingdom). The anatomical scan used an inversion recovery 3-D T1-weighted gradient-echo sequence images (TE = 4.3 ms, TR = 11.2 ms, TI = 400 ms, matrix = 384 × 384, slice thickness = 1.2 mm). Functional images were acquired using a gradient echo planar (EPI) sequence (TE = 30 ms, TR = 2000 ms, flip angle = 90°, FOV = 260 × 260, matrix = 64 × 64, slice thickness = 3 mm, 42 contiguous sections, in plane resolution 4.06 mm). The first four volumes of each functional run were discarded in order to allow longitudinal magnetization to approach equilibrium.

#### Pre-processing of fMRI data

All data were processed using SPM5 software (Statistical Parametric Mapping 5, Welcome Dept. Cognitive Neurology, UK^1^). Standard pre-processing procedures were applied to MRI data. EPI volumes were corrected for slice timing, realigned to the first image, co-registered with the high-resolution T1-weighted image and normalized into the Montreal Neurological Institute (MNI) template. Finally, the normalized EPI volumes were smoothed using an isotropic Gaussian kernel filter of 5 mm full-width half-maximum.

#### Statistical analysis of fMRI data

Only cerebral activity for responses meeting criteria for the self and imagery conditions were used for the subsequent analyses. A trial was considered as a hit if (1) the participant had pressed the button during the trial (indicating retrieval) and (2) the description of the memory recalled in the scanner during the debriefing met the criterion for SAM or the description of scene imagery in the scanner during the debriefing met the criterion for non-self reference. Memory retrieval was modeled by convolving the time period between cue presentation and subjects’ response with the hemodynamic response function (HRF). For each subject, the General Linear Model was used to estimate the parameters of interest. Parameters of movement were also included in the model as regressors of no interest.

Whole brain *t*-tests were computed to estimate the contrast of interest for each subject: self vs. rest, and imagery vs. rest.

Individual contrasts were entered in a second level analysis using a 2 groups (young-old) × 2 conditions (self-imagery) factorial design. A corrected (FWE) cluster level threshold of *p* < 0.05 was used (voxel-wise threshold was set at *p* < 0.001 uncorrected).

#### Regression analyses

We employed the multiple regressions model in SPM to test the link between brain activations and behavioral performance. We entered in the model the contrast between the self and the imagery condition for each subject and the CRI and CRS scores. To improve statistical power we ran a first analysis on the whole group of subjects (young and older). The threshold was set at *p* < 0.001 uncorrected at voxel level, and reported results survived small volume correction (SVC) at p(FDR) <0.05. SVC correction was used since we looked for an effect only in areas showing either a main effect of condition or a main effect of group.

We then extracted signal change in the region showing a correlation with MarsBar (Brett et al., 2002) for each subject, and ran mediation analysis using partial correlation with STATISTICA7© to investigate the effect of age on the link between brain activation and memory performances.

## Results

### Behavioral results

According to the debriefing and the responses in the scanner, both young and older participants showed a similar high percentage of analyzable trials (self condition, for YA: mean 91.74% ± 1.08, OA: mean 86.71% ± 1.65, *t*_(33)_ = 1.62, *p* = 0.11; imagery condition, for YA: mean 92.46% ± 0.06, OA: mean 87.89 ± 0.08, *t*_(33)_ = 1.81, *p* = 0.08) and a similar rapid response time for both conditions after time presentation of cue, i.e., 5 s (self, for YA: mean 2.27 s ± 1.08, OA: mean 2.93 s ± 1.65, *t*_(33)_ = −1.40, *p* = 0.17; imagery, for YA: mean 2.0 s ± 1.07, OA: mean 2.31 ± 1.27, *t*_(33)_ = −0.77, *p* = 0.44).

We compared the percentages of CRI and CRS in two mixed ANOVAs with groups (YA vs. OA) as a between-groups factor and encoding conditions (self vs. imagery) as a within-subjects factor.

#### CRI

**Figure 1:**
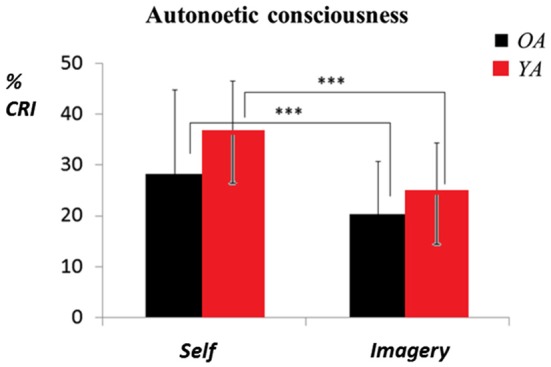
Mean percentages (and standard deviations) of correctly recollected items (CRI) by group and encoding condition (effect of condition, ***p < 0.001, Bonferroni correction).

Figure 1 shows the percentages of CRI by group and encoding condition.

The analysis revealed no effect of group, *F*_(1,33)_ = 2.44, *p* = 0.13, $\eta_{p}^{2}$ = 0.06, revealing no significant difference between YA and OA (30.40% vs. 24.30%). A main effect of encoding condition, *F*_(1,33)_ = 15.68, *p* = 0.0003, $\eta_{p}^{2}$ = 0.31, indicated that participants recollected more self-referenced items than items encoded in the control condition (32.36% vs. 22.34%). Finally, there was no significant interaction, *F*_(1,33)_ < 1 (see Figure 1). Of note, there were no Guess responses regardless of the group and the condition and very few Know responses (YA: mean 1.78% ± 4.69, OA: mean 0.22% ± 0.95, *t*_(33)_ = −1.52, *p* = 0.14).

#### CRS

**Figure 2:**
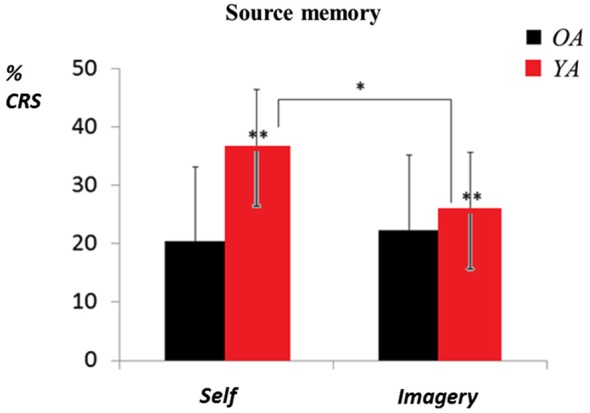
Mean percentages (and standard deviations) of correctly recalled sources (CRS) by group and encoding condition (effects of group and condition,* *p* < 0.05, ** *p* < 0.01, Bonferroni correction).

Figure 2 shows the percentages of CRS by group and encoding condition.

The analysis revealed a main effect of group, *F*_(1,33)_ = 9.04, *p* = 0.004, $\eta_{p}^{2}$ = 0.20, showing that OA produced fewer correct responses than YA (21.34% vs. 31%). There was no effect of encoding condition, *F*_(1,33)_ = 2.57, *p* = 0.12, $\eta_{p}^{2}$ = 0.06 (*self vs. imagery*: 28.38% vs. 23.96%), but a significant interaction between group and encoding condition, *F*_(1,33)_ = 5.31, *p* = 0.02, $\eta_{p}^{2}$ = 0.13 (see Figure 2). Subsequent comparisons using Bonferroni *post hoc* tests showed that for OA, self-reference had no effect on the percentages of correct responses (20.38% vs. 22.30%, *p* = 0.96), whereas the difference was significant for YA (36.37% vs. 25.62%, *p* = 0.04). Moreover, there was no difference in performance in the *imagery* condition (*p* = 0.86), but the performance of the YA group was significantly better than that of the OA group in the *self* condition (*p* = 0.001).

### fMRI data

#### Encoding

**Figure 3:**
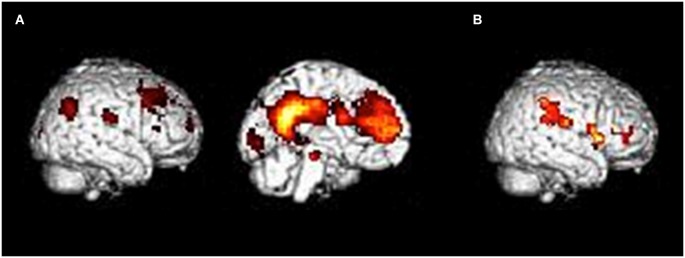
**Results of the ANOVA with group (young-old) and condition (self-imagery) as factors. (A)** Main effect of condition. All regions were more activated during self, compared to the imagery condition. **(B)** Main effect of group. All regions were more activated in the older, compared to the young group. Results are superimposed to a single subject rendering and are significant at a cluster level p(FWE) < 0.05.

The ANOVA revealed a main effect of condition in a large cluster encompassing frontal and posterior medial structures, including the MPFC and the precuneus/PCC. Moreover, lateral parietal regions and visual areas were activated. Finally, activations in the left parahippocampal gyrus and hippocampus were reported (see Figure 3A). All these regions were more activated in the self condition. The list of local activation maxima is reported in Table 1. We also report a main effect of group in a fronto-parietal network encompassing inferior frontal and parietal structures (see Figure 3B). These regions were more activated in the older group. The list of local activation maxima is reported in Table 2. We did not observe any regions showing an interaction between the two factors.

**Table 1:** **Main effect of condition**.

Label	probable BA	*k*	*t*	*z*	MNI coordinates		
					*x*	*y*	*z*
L PCC	31	5755	61.93	6.48	−6	−51	24
L MCC	31		57.69	6.31	−12	−42	33
L Mid. Front. Gyrus	8		52.66	6.09	−21	27	36
L Sup. Medial. Front. Gyrus	9		35.34	5.17	0	45	27
L Ang. Gyrus	39	272	46.05	5.77	−45	−69	33
L Parahipp. Gyrus		142	34.88	5.14	−15	−27	−15
L Hipp.			32.53	4.99	−27	−21	−18
R Calcarine Gyrus	17	174	31.72	4.94	12	−93	9
R Cuneus	18		23.83	4.34	12	−90	18
L Superior. Occ. Gyrus	17		20.57	4.06	−12	−93	3
R Ang. Gyrus	39	117	21.85	4.17	51	−66	36
R Inf. Par. Gyrus	39		15.34	3.52	42	−57	36

*L = left; R = right; PCC = posterior cingulate cortex; MCC = middle cingulate cortex; Mid. Front. Gyrus = middle frontal gyrus; Sup. Medial. Front. Gyrus = superior medial frontal gyrus; Ang. Gyrus = angular gyrus; Parahipp. Gyrus = parahippocampal gyrus; Hipp. = hippocampus; Superior. Occ. = superior occipital gyrus; Inf. Par. Gyrus = inferior parietal gyrus. All reported activations are significant at a cluster threshold of p(FWE) < 0.05*.

**Table 2:** **Main effect of group**.

Label	probable BA	*k*	*t*	*Z*	MNI coordinates		
					*x*	*y*	*z*
R Inf. Front.	44	215	24.05	4.36	48	6	12
Gyrus
R Insula			23.45	4.31	33	30	9
R Putamen			19.24	3.93	33	9	6
R Inf. Par. Gyrus	40	201	20.72	4.07	57	−36	33
R Inf. Par. Gyrus	40		17.82	3.79	63	−30	24

*L = left; R = right; Inf. Front. Gyrus = inferior frontal gyrus; Inf. Par. Gyrus = inferior parietal gyrus. All reported activations are significant at a cluster threshold of p(FWE) < 0.05*.

#### Regression analyses

**Figure 4:**
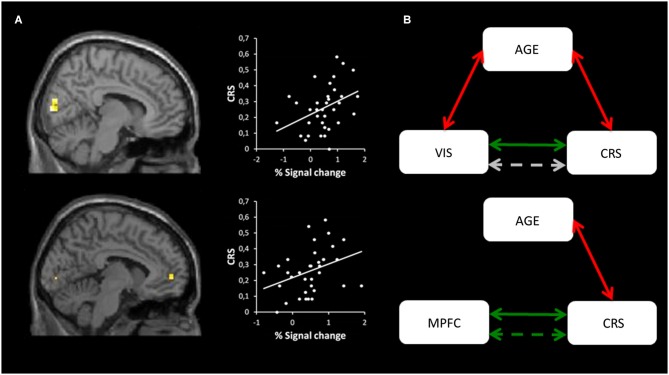
**Multiple regressions and mediation analysis. (A)** The first column shows the regions showing a correlation with CRS scores: Cuneus and MPFC are shown in the upper and lower part of the figure respectively. Statistical maps are superimposed to an MNI T1 template. Statistical threshold was set at *p* < 0.001 (uncorrected) and survived SVC with a p(FDR) <0.05. The second column shows the graphical representation of the correlation between the CRS (y-axis) and the Cuneus activation (x-axis), and the CRS and the MPFC activation (x-axis) respectively on the upper and lower part of the figure. **(B)** Mediation analysis showing correlation between visual areas (Cuneus) and CRS before (green line, *r* = 0.38, *p* = 0.02) and after (gray dotted line, *r* = 0.24, *p* = 0.17) removing the effect of age in the upper part of the figure. In the lower part, the same mediation analysis shows that the correlation between MPFC and CRS (green line, *r* = 0.35, *p* = 0.04) remains significant after correcting for age (green dotted line, *r* = 0.35, *p* = 0.04). Red arrows represent negative correlations between Cuneus activation and age (*r* = −0.39, *p* = 0.02), and CRS and age (*r* = −0.49, *p* = 0.003).

Multiple regressions revealed a link between CRS and visual areas and between CRS and the MPFC (see Figure 4A), for the coordinates of regions showing correlations with CRS see Table 3. No correlations were reported for the CRI score. Partial correlations showed that correlations with the visual areas were no longer significant after controlling for the age of the subjects, while correlation with the MPFC remained unchanged (see Figure 4B).

**Table 3:** **Regression**.

Label	probable BA	*k*	*t*	*Z*	MNI coordinates		
					*x*	*y*	*z*
R Cuneus	17	40	5.10	4.33	9	−90	6
R Cuneus	18		4.86	4.17	12	−87	24
L Med. Front. Gyrus	10	7	4.21	3.72	−6	54	0
L Midd. Occ. Gyrus	18	13	4.21	3.72	−24	−93	3

*L = left; R = right; Med. Front. Gyrus = medial frontal gyrus; Midd. Occ. Gyrus = middle occipital gyrus. Reported activations are significant at p(FDR) < 0.05 using a small volume correction (SVC)*.

## Discussion

Our study aimed to explore the neural correlates of the SRE via autobiographical memory and their age-related changes. In particular, for the first time we explored the relationship between the neural bases of incidental self-referencing encoding via SAM and qualitative features of subsequent episodic memory in a group of young and older adults. We found age-related differences in the effect of SAM compared to general semantic imagery on subsequent episodic memory retrieval.

At the behavioral level, we showed that the particular self-referential task tapping SAM is an effective encoding strategy since it improves memory performance compared to general semantic encoding (see Klein et al., 1989; Symons and Johnson, 1997; Klein, 2012). Very interestingly, we demonstrated that compared to a control condition that necessitated cognitive processes shared by SAM, such as accessing stored general semantic knowledge and mental scene construction, but with no reference to personal information, the self-referential encoding still produced a memory improvement. This confirms that self-referential processes have particular mnemonic properties which are more efficient than deep semantic processing. This experiment extends previous findings by revealing the robustness of self-reference on subsequent episodic memory, even by using incidental encoding and delayed free recall.

Remarkably, despite the task difficulty (incidental encoding and free recall), that could have been unfavorable for the elderly, the SRE seems to be partially preserved in aging. Indeed, our behavioral results indicate that older adults’ recollection via autonoetic consciousness is enhanced for self-referenced information. This is in line with previous studies showing that the SRE boosts the ability to remember previous events in a subjective way in young (Conway and Dewhurst, 1995; Lalanne et al., 2013) as well as in older adults (Kalenzaga et al., 2012; Kalenzaga and Clarys, 2013; Lalanne et al., 2013). Thus, the self-referential processes that remain intact in aging are efficient enough to enhance delayed free recall of (incidentally encoded) items and subjective recollective experience. Nevertheless, contrary to Hamami et al. (2011) and Dulas et al. (2011), we failed to show a benefit on source memory for older adults. Hamami et al. (2011) used very dissimilar encoding conditions (i.e., self-reference vs. commonness and vs. structural judgment), and Dulas et al. (2011) used judgments of pleasantness as a self-reference condition, and commonness discrimination as a control condition. Thus, it is possible that the difference in the tasks may explain the difference in results. In our case, the self-reference and the control condition shared several key processes (see above). Moreover, we tested detailed source memory that referred to recalling the condition of encoding (self vs. imagery), how the item had been processed (e.g., internally-generated context such as pressing the button and accessing personal or impersonal scene imagery). Thus, in accordance with previous studies (Hashtroudi et al., 1989; Henkel et al., 1998), we can hypothesize that the similarity between the two experimental tasks could have made source retrieval more difficult for older adults. Moreover, binding of perceptual (verbal cue), temporal, semantic, and affective properties, as well as information about the cognitive operations that took place at the time of the cue-based task may explain older adults’ difficulty in retrieving the encoding context (Johnson et al., 1995).

Probably the most important aspect in explaining our findings is that we used delayed free recall which is a highly demanding task, especially for elderly people, while other studies used source memory after “old-new” recognition. This is in line with some recent findings showing that compared with alternative encodings, self-reference incidental encoding significantly enhances delayed memory performance on free recall in young adults but not in older adults, while it enhances recognition in both populations (Lalanne et al., 2013). Free recall engages more self-initiated processes to recover the original encoding situation than recognition, and requires a great amount of cognitive resources which are deficient in normal aging (Craik, 1986; Naveh-Benjamin and Old, 2008). Finally, the SRE does not seem sufficient to help older adults to efficiently use multiple distinctive characteristics of items and sources in delayed free recall (i.e., what, how and when sources) (see Ferguson et al., 1992). Overall, our behavioral results revealed a behavioral dissociation in healthy aging between autonoetic consciousness, improved by self-referential processing, and source memory, that does not benefit from self-reference.

At the neural level, the contrast between the self-reference (SAM) and the control condition showed a similar pattern of activation in young and older adults. This similarity was further confirmed by a conjunction analysis testing for convergent activation across the two groups. We basically reported activations in the same regions showing a main effect of condition in the ANOVA, in particular in the MPFC, PCC/precuneus and angular gyrus (Supplementary Table 1). Activations across the two groups mainly concerned CMS, namely the network involved in self-referential processing, such as the MPFC, PCC, MCC, cuneus and precuneus (Northoff et al., 2006; Denny et al., 2012; Martinelli et al., 2013c). All these regions have been previously associated with mentalizing about self-concepts such as goals, beliefs, and perceptions of oneself (Northoff et al., 2006; Denny et al., 2012). Hence, we found a pattern of activation quite similar to that found in young and older adults during a self-referential task tapping conceptual self-knowledge (Macrae et al., 2004; Gutchess et al., 2007, 2010; Zhu et al., 2012). Moreover, we confirmed that the cerebral network devoted to the self is preserved in healthy aging. No significant difference appeared in this network when the two age groups were directly compared. We only reported a greater activation in fronto-parietal areas in older adults, but independently of the condition. Attentional control is generally associated with a network of frontal and parietal regions (e.g., Kastner and Ungerleider, 2000; Corbetta and Shulman, 2002; Spreng et al., 2010). As young and older groups performed equally on SAM and control tasks (see the results from debriefing), over-recruitment of a fronto-parietal network in the elderly, when accompanied by similar behavioral performances can be considered to be compensatory (Cabeza et al., 1997; Reuter-Lorenz and Cappell, 2008; Campbell et al., 2012). Thus, our findings demonstrate that activation in the cerebral network responsible for self-referential processes is comparable between young and older adults during the incidental encoding of self-referential information, and suggest that activity in this network boosts the subsequent recollection.

In order to explore the relationship between the regions activated by the self vs. imagery contrast at encoding and the SRE, we conducted regression analyses within the whole population of subjects. Links were noted between source memory (CRS) and both the MPFC (BA 10) and visual areas during SAM condition. In other words, the more participants (regardless of their age) activated these structures at encoding, the better their subsequent episodic source memory performance. The correlation with the activity in the MPFC at encoding is in line with previous studies underlining its role in successful SRE in episodic memory (Macrae et al., 2004; Zhu et al., 2012). More specifically, this is consistent with Leshikar and Duarte (2012) study reporting that the activity of the MPFC (BA10) at encoding in a self-reference condition is predictive of source memory accuracy in young adults. In the same vein, a recent study in young adults found that the SRE on subsequent recognition was associated with greater activity in the MPFC during the encoding phase (not during the retrieval phase) (Morel et al., 2014). The present study expands the finding on the role of this region on the SRE to tasks tapping SAM. This demonstrates a positive effect of the reactivation of self-related generic memories on the encoding of new information in episodic memory.

As far as older adults were concerned, the finding confirms previous studies indicating that the activity of the MPFC in the SRE is preserved in healthy aging (Gutchess et al., 2007), thus seemingly arguing in favor of a preservation of functionality of this region in older adults. However, the findings also reveal a correlation between CRS and the activity in the visual areas during the SAM condition which was mediated by age. The relationship between mental imagery and recollection has already been emphasized (Dewhurst and Conway, 1994). Donix et al. (2010) showed a relative age-related increase in the activity of posterior brain regions when recalling autobiographical memories, and suggested that it could reflect changes in visuospatial processing during episodic memory retrieval in older adults. Impairment in mental imagery in older adults is supported by behavioral studies that reveal a decline in the generation, maintenance, and manipulation of mental images (Dror and Kosslyn, 1994; De Beni et al., 2007; Brewer and Barton, 2012). Moreover, Kalkstein et al. (2011) established that aging disrupts neural networks that subserve mental imagery and offered evidence that it is a factor of age-related memory decline (Palladino and De Beni, 2003). Thus, it is possible that abilities based on the reactivation of self-related mental imagery at encoding play a pivotal role in source memory of self-related items. The very specific aspects of self-referential processes (MPFC) may not be salient enough in aging, requiring over-recruitment of other structures to promote the SRE on source memory accuracy. Thus, our data suggest a relative differential functionality of the MPFC in young and older adults, with a lower specificity in aging in areas related to successful encoding of self-referenced information. Overall, it may support the idea of an age-related functional reorganization in the neural networks underlying self-related long-term memory of new items (Gutchess et al., 2010).

Nevertheless, further research is needed to substantiate our findings in aging. Indeed, the absence of the SRE on source memory in older adults could result from the relatively small sample size and the experimental design tapping SAM instead of more abstract self-knowledge. In fact, our experimental self-referencing task was more difficult than just making judgments about self-traits. In this line, the over-recruitment of the attentional/fronto-parietal network in older adults to accomplish the tasks in the scanner must be considered. Moreover, incidental encoding and free recall are particularly difficult for the elderly. Future neuroimaging research in aging should compare different kinds of self-referencing processes on the subsequent recall and recognition of items and source memory, using a large sample size.

In conclusion, the aim of the present study was to test the effectiveness of self-referencing as a strategy to support episodic memory encoding in young and older adults using an original self-reference task tapping generic autobiographical memories. Our study shows that the regions engaged during self-referential processing are resilient in the elderly, but highlights age differences in the processes related to successful encoding of self-referenced items. First, though self-referencing improves subjective recollection in young and older adults, it has an effect on source memory performance in young adults only. Moreover, though the activity of the MPFC engaged during self-referential processing improves the source memory of self-related items regardless of age, the activity of visual regions also improves the source memory of self-related items in aging. The present findings may reveal some differences in the functionalities of the MPFC regarding the very specific aspects of self-reference on episodic memory in aging.

## Conflict of interest statement

The authors declare that the research was conducted in the absence of any commercial or financial relationships that could be construed as a potential conflict of interest.

## Footnotes

^1^www.fil.ion.ucl.ac.uk/spm
