# Supplementary material for: Episodic memory and self-reference via semantic autobiographical memory: insights from an fMRI study in younger and older adults
Source: Front Behav Neurosci. 2015 Jan 13;8:449. doi: 10.3389/fnbeh.2014.00449 (PMC4292587; doi:10.3389/fnbeh.2014.00449)
Supplement: Supplementary file 1 [file Table_1.DOCX]

**Supplementary Table** Conjunction between Young and Old subjects for the contrast self *vs* imagery.

|  |  | |  | |  |  | **MNI coordinates** | | |
| --- | --- | --- | --- | --- | --- | --- | --- | --- | --- |
| **Label** | | **probable BA** | | **k** | **t** | **z** | **x** | **y** | **z** |
| L PCC | 31 | | 578 | | 5.33 | 4.85 | -6 | -51 | 21 |
| L PCC | 31 | |  | | 5.19 | 4.73 | -12 | -42 | 33 |
| L Precuneus | 31 | |  | | 4.75 | 4.39 | -15 | -60 | 21 |
| L Ang. Gyrus | 39 | | 70 | | 4.56 | 4.24 | -45 | -69 | 33 |
| L Mid. Front. Gyrus | 10 | | 186 | | 3.98 | 3.75 | -9 | 48 | 6 |
| R Mid. Front. Gyrus | 9 | |  | | 3.85 | 3.64 | 3 | 45 | 24 |
| R ACC | 24 | |  | | 3.84 | 3.64 | 6 | 33 | 15 |

L=left; R=right; PCC=posterior cingulate cortex; Ang. Gyrus= angular gyrus; Mid. Front. Gyrus=middle frontal gyrus; ACC=anterior cingulate cortex. All reported activations are significant at a cluster threshold of p(FWE)<0.05.
